# Involvement of Mesenchymal Stem Cells in Oral Mucosal Bacterial Immunotherapy

**DOI:** 10.3389/fimmu.2020.567391

**Published:** 2020-11-19

**Authors:** Alberto Vázquez, Lidia M. Fernández-Sevilla, Eva Jiménez, David Pérez-Cabrera, Rosa Yañez, Jose Luis Subiza, Alberto Varas, Jaris Valencia, Angeles Vicente

**Affiliations:** ^1^Department of Cell Biology, School of Medicine, Complutense University of Madrid, Madrid, Spain; ^2^Instituto de Investigación Sanitaria, Hospital Clínico San Carlos, Madrid, Spain; ^3^Hematopoietic Innovative Therapies Division, Centro de Investigaciones Energéticas, Medioambientales y Tecnológicas, Centro de Investigación Biomédica en Red de Enfermedades Raras, Instituto de Investigaciones Sanitarias de la Fundación Jiménez Díaz, Madrid, Spain; ^4^Inmunotek, Alcalá de Henares, Madrid, Spain

**Keywords:** mesenchymal stem cells, sublingual mucosal immunotherapy, polybacterial preparation, vaccine, immunomodulation, pattern recognition receptors, short-term memory

## Abstract

Recent clinical observations indicate that bacterial vaccines induce cross-protection against infections produced by different microorganisms. MV130, a polyvalent bacterial sublingual preparation designed to prevent recurrent respiratory infectious diseases, reduces the infection rate in patients with recurrent respiratory tract infections. On the other hand, mesenchymal stem cells (MSCs) are key cell components that contribute to the maintenance of tissue homeostasis and exert both immunostimulatory and immunosuppressive functions. Herein, we study the effects of MV130 in human MSC functionality as a potential mechanism that contributes to its clinical benefits. We provide evidence that during MV130 sublingual immunization of mice, resident oral mucosa MSCs can take up MV130 components and their numbers remain unchanged after vaccination, in contrast to granulocytes that are recruited from extramucosal tissues. MSCs treated *in vitro* with MV130 show an increased viability without affecting their differentiation potential. In the short-term, MSC treatment with MV130 induces higher leukocyte recruitment and T cell expansion. In contrast, once T-cell activation is initiated, MV130 stimulation induces an up-regulated expression of immunosuppressor factors in MSCs. Accordingly, MV130-primed MSCs reduce T lymphocyte proliferation, induce the differentiation of dendritic cells with immunosuppressive features and favor M2-like macrophage polarization, thus counterbalancing the immune response. In addition, MSCs trained with MV130 undergo functional changes, enhancing their immunomodulatory response to a secondary stimulus. Finally, we show that MSCs are able to uptake, process and retain a reservoir of the TLR ligands derived from MV130 digestion which can be subsequently transferred to dendritic cells, an additional feature that also may be associated to trained immunity.

## Introduction

Recurrent respiratory tract infections (RRTIs) are a leading cause of morbidity and mortality in children and adults (1, 2). While many of the RRTIs are of viral origin, antibiotics are often misused in these conditions leading to bacterial resistance and microbiota disruption (3–5). Therefore, implementation of effective strategies to improve their management has become a therapeutic challenge (6). An increasing number of studies are focused on prophylactic and therapeutic interventions that enhance the body’s natural defenses against infections and/or downregulate the accompanying harmful inflammatory process (2, 7). Mucosal immunotherapy with bacteria-derived products or whole cell bacteria may play that role. It has been shown that poly-bacterial preparations (PBP) improve RRTIs in both adults and children by reducing the number, duration and severity of the clinical episodes (7–11). Mucosal bacterial immunotherapy induces a broad range of both non-specific and specific immune responses in mucosal and extra-mucosal tissues (7, 12–14). Immune mechanisms include the induction of antimicrobial peptide and antiviral cytokine release, neutrophil and monocyte recruitment and also the modulation of the adaptive response, mainly through its effects on dendritic cells (DCs) (2, 13, 15, 16).

MV130 is a sublingual PBP that contains different species of inactivated whole- cell Gram-positive and negative bacteria (17). MV130 significantly reduces the patient infection rate inducing both a specific T cell immunity against bacteria included in MV130 and an enhancement in T cell responses to unrelated antigens (7, 9, 11, 14). MV130 triggers Toll-like receptors (TLR) and Nod-like receptors (NLR), imprints human DCs with the capacity to generate Th1 and Th17 responses and increases the IL-10 cytokine levels (17). Bacterial-derived products may also act activating non-immune cells such as mucosal epithelial cells and mesenchymal stem cells (MSCs) (18, 19). Although bone marrow, adipose tissue, and umbilical cord blood are the prevailing sources of MSCs used in cell therapy for its greater availability, these cells represent a naturally heterogeneous cell population that can be isolated from a wide range of tissues. Relevant in the context of sublingual delivery of vaccines or PBP, MSCs have been described in human oral soft tissues including oral mucosa proper, gingiva, periodontal ligament, dental follicle and dental pulp (20, 21). In addition, recent studies have reported the presence of numerous MSCs in fetal and adult connective tissue from human major salivary glands, including parotid, sublingual, and submandibular glands (22, 23). These oral MSCs show phenotypical and functional resemblance to MSCs isolated from other tissues (20).

In contrast to the therapeutic role of systemically delivered MSCs, the role of local resident MSCs during infection and the subsequent immune response is unclear at present. Different studies indicate that tissue-resident MSCs could function as early sensors of pathogens when classical immune cells have not been recruited yet, since MSCs are equipped with a wide set of pattern recognition receptors (PRRs), mainly TLRs (24). After TLR-triggering or stimulation with inflammatory cytokines, MSCs acquire a cell autonomous, broad-spectrum antimicrobial effector function directed against clinically relevant bacteria, protozoan parasites and viruses (25–27). Together with these direct effects, MSCs may interact with innate immune cells recruited at the inflammation site, and then, their function would be modulated to establish a fine balance between pathogen clearance and repair processes (28). This balance is essential for controlling inflammation, preserving tissue homeostasis, and preventing organ failure (28–30). Mechanisms triggering a functional switch between pro-inflammatory and anti-inflammatory MSC phenotypes include dose, duration and type of TLR stimuli, as well as expression levels of different autocrine and paracrine pro- and anti-inflammatory cytokines (31–33). MSCs may also act indirectly, driving the polarization of the functional phenotype of immune cells (31–35). In addition, it has been recently suggested that MSCs could be primed by certain pathogens, which would increase their response to a second stimulus, displaying therefore a trained immunity similarly to innate immune cells (18, 36–38). Thus, MSCs could be able not only to dampen the inflammatory response but also to enhance bacterial clearance, as has been demonstrated in preclinical models of sepsis, bacterial pneumonia, and acute respiratory distress syndrome (39–43).

In the present study we report the main effects of MV130 on MSC biology and the impact in their immunoregulatory features. Our data provide first evidence of mechanisms that might be involved in the observed clinical benefits of MV130.

## Material and Methods

### Culture of Human Mesenchymal Stem Cells (MSCs)

Human bone marrow MSCs from healthy donors (*n* = 8; Innoprot) were cultured in Mesenpro medium (Thermo Fisher Scientific) supplemented with glutamine and penicillin-streptomycin (Lonza) in a humidified incubator at 37°C with 5% CO_2_ until reaching a confluence of 80%. Half of the culture medium was renewed every 3–4 days. MSCs were used between 3rd 7th passages.

### MSC Treatments

MV130 (Bactek^®^, Inmunotek S.L. Spain), is a preparation of whole-cell heat-inactivated bacterial species including 90% Gram-positive bacteria (*Streptococcus pneumoniae*, *Staphylococcus aureus*, S*taphylococcus epidermidis*) and 10% Gram-negative bacteria (*Klebsiella pneumoniae*, *Moraxella catarrhalis*, *Haemophilus influenzae*).

MSCs were treated with MV130 (10^7^ bacteria/mL; MV130-MSCs) for different time periods as indicated in each section. Where indicated, other concentrations of MV130 were used. After treatment, culture medium was removed and cells were washed twice with warm PBS to completely remove the unbound bacterial preparation. MSCs under control conditions were treated with the same volume of the excipient of MV130 (CTRL-MSCs).

To analyze if MSCs primed with MV130 during 24 h modify their response to a second challenge, MSCs were gently washed with warm PBS and cultured for another 3 days. Then, cells were re-stimulated with IFNγ (2 ng/mL; Immunotools) for another 24 h. Supernatants were collected and cells were used to carry out migration assays or to perform co-cultures with T cells (see scheme below).

**Scheme 1:**
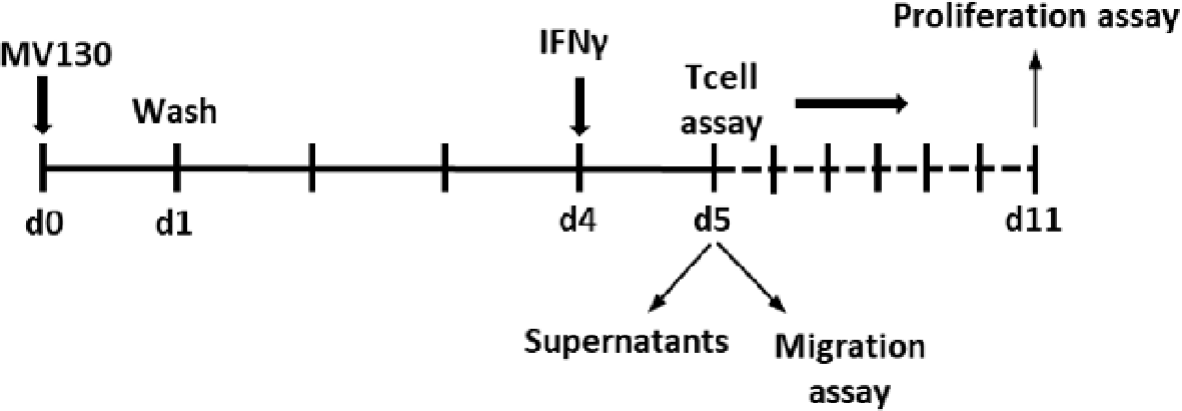


### MV130 Staining With CFSE (CFSE-MV130)

Bacteria from MV130 were stained with CFSE (Biolegend) at 2.5 µM following the manufacturer’s instructions, to monitor their presence by immunofluorescence or flow cytometry.

### Differentiation Assays

MSCs were cultured at a cell density of 5,000 cells/cm^2^ in 24-well culture plates for 6 days. MSCs were treated with MV130 (10^7^ bacteria/mL) or excipient and this treatment were repeated once again 24 h later. 72 h after the last dose of MV130, cells were collected, and expression of *NANOG* and *Oct-4* was analyzed by quantitative PCR. In parallel, other cultures were switched to a specific adipogenic or osteogenic conditioning medium, as described previously (44). For osteogenic differentiation, alkaline phosphatase enzyme (ALP) levels were determined as a measure of MSC differentiation into osteoblasts, after 5 days of culture, while for adipogenic differentiation, Oil Red staining (ORO) was performed 10 days after differentiation conditions.

### PBMC, Monocyte, and T Cell Isolation

Peripheral blood mononuclear cells (PBMCs) were obtained by density gradient centrifugation using lymphocyte isolation solution (Rafer) from buffy coats of volunteer healthy donors (Centro de Transfusión de la Comunidad de Madrid, Spain). Monocytes were obtained from PBMCs by immunomagnetic isolation using anti-CD14 microbeads and VarioMACS cell separator (Miltenyi Biotec), following the manufacturer’s protocol. Non-adherent T lymphocyte-enriched cell suspensions were obtained from PBMCs by nylon wool enrichment and labeled with CFSE (Biolegend) at 2.5 µM following the manufacturer’s instructions. The percentage of CD3^+^ cells were always above 90%.

### MSC-Monocyte Co-Cultures

MSCs were seeded in 6-well plates at a concentration of 5 × 10^5^ cells/well in 2 mL. Following overnight adherence, MV130 treatment was added during 24 h, and then MSC cultures were gently washed to remove the unbound bacteria. Monocytes were added at 1:10 MSC/monocyte ratio in the different conditions described below.

### Monocyte-Derived DCs and M1 Macrophages

Monocytes were cultured in RPMI 1640 medium (Lonza) supplemented with 10% fetal bovine serum (Gibco, Thermo Fisher Scientific), 100 U/mL penicillin, 100 µg/mL streptomycin, 2 mM L-glutamine and 1 mM pyruvate (all from Lonza), referred to as complete-RPMI medium, in the presence of GM-CSF (5 ng/mL; Gibco, Thermo Fisher Scientific) to induce M1 macrophages or with GM-CSF (20 ng/mL) and IL- 4 (20 ng/mL; Gibco, Thermo Fisher Scientific) to induce DC differentiation. After 3 days, additional 5 ng/mL GM-CSF was added to macrophage cultures and half of the medium was renewed in DC cultures. At day 6 of co-culture, macrophage and DC phenotypes were analyzed by flow cytometry within CD90^-^ population.

Macrophages and DCs were stimulated overnight with LPS (Invitrogen, Life Technologies) at 10 ng/mL or 50 ng/mL, respectively. Supernatants were collected and their allostimulatory function was analyzed by culturing in mixed lymphocyte reaction (MLR) with CFSE-labeled T lymphocytes (1:10 Macrophage or DC/T cell ratio). After 5 days of co-culture, T lymphocyte proliferation was analyzed using the CFSE dilution method by flow cytometry in the CD3^+^ population. Supernatants from different co-cultures were harvested at different times and cytokine secretion was measured.

### MSC-T Cell Co-Cultures

2.5 × 10^3^ MSCs were seeded in duplicate and allowed to adhere to 48-well plates for 12 h and after that, primed with MV130 or under control conditions for 24 h. After treatment, MSCs were gently washed and CFSE-T lymphocytes were seeded at 1:25 MSC/T cell ratio in warm complete-RPMI with Dynabeads Human T-Activator CD3/CD28 for T Cell Expansion and Activation (1:4 Bead/T cell ratio; Gibco Thermo Fisher Scientific). Stimulated and non-stimulated T lymphocytes cultured without MSCs was carried out as control. After 3, 4 or 5 days of co-culture, supernatants were collected and proliferation of CFSE-T lymphocytes (CD3^+^ cells) and CD69 expression were analyzed by flow cytometry.

### Mice

Mice were housed in the animal facility (Registration No. ES280790000183) at CIEMAT (Madrid, Spain). Mice were routinely screened for pathogens in accordance with FELASA procedures and received water and food *ad libitum*. All experimental procedures were carried out according to Spanish and European regulations (Spanish RD 53/2013 and Law 6/2013, European Directive 2010/63/UE). Procedures were approved by the CIEMAT Animal Experimentation Ethical Committee according to approved biosafety and bioethics guidelines.

### MV130 Sublingual Administration

Male mice C57 (3 weeks old) were sublingually treated with MV130 (10^9^ bacteria/mL) or excipient (control group) in two consecutive doses of 10 µL. This was repeated for 5 consecutive days with a booster two days later and 24 h before sacrifice (see scheme below). Sublingual administration was performed under anesthesia (mixture of 5% isoflurane in oxygen), to ensure proper delivery and prevent swallowing.

Peripheral lymph nodes (P-LN), submaxillary lymph nodes (SM-LN) and oral mucosa (OM) were excised and processed for flow cytometry. Lymph node cell suspensions were obtained by gentle mechanical disruption with a potter homogenizer until completely disaggregated. Oral mucosa cell suspensions were obtained by enzymatic digestion for 1 h with DNase (100 µg/mL), dispase (800 µg/mL), and collagenase (20 µg/mL) (Roche). Cells were stained with CD45, CD3, F4/80, CD29, MHC-II, and CD19 mAbs. Cells were gated based on forward/side scatter characteristics and their ability to exclude propidium iodide. Leukocyte populations were analyzed according to CD45^+^ expression and, CD3^+^ for T lymphocytes, F4/80^+^MHC-II^lo^CD19^-^ for macrophages and F4/80^-^MHC-II^+^CD19^-^ for dendritic cells. MSCs were identified as CD45^-^CD29^+^Sca-1^+^ cells. For tissue histological analysis, oral mucosa was carefully removed, embedded in OCT compound (Thermo Fisher Scientific) and stored at -80°C until processing. Sections of 10 μm were blocked with 5% normal donkey serum, following the staining with anti-mouse CD45 and Hoechst 33342 (Invitrogen, Life Technologies) for staining the nuclei. For immunofluorescence analysis, preparations were mounted using FluorSave (Millipore) and imaged using a fluorescence microscope (Nikon Eclipse Ci) with a digital camera (Nikon DS-U3) and Nis-Elements D software. Images were assembled using ImageJ software.

### LPS-Induced Inflamed Pad Mouse Model

FVB/NJ mice, sedated with isoflurane, received a single injection of 40 μg of LPS in 30 µL of PBS into the right pad. At the same time, 30 μL of PBS were injected into the left pad as control. The baseline measurement was determined by measuring the pad thickness of each mouse with a digital caliper before LPS administration. 24 h after LPS injection, 5 × 10^5^ MSCs, treated as described above, were intravenously infused through the tail vein. To assess the efficacy of the different experimental groups of MSCs, the right pad thickness was measured 24, 48, and 72 h after LPS administration, comparing it to the control pad. At the end of the experiments mice were sacrificed by CO_2_ inhalation. Footpads were extracted and processed for histological or flow cytometry analysis. Cell suspensions were obtained by gentle mechanical disruption with a potter homogenizer until completely disaggregated and were stained with CD45, CD3, F4/80 and Gr-1 mAbs to identify T-cells, inflammatory and classic macrophages, and granulocytes (45). For histological analysis, pad samples were fixed in buffered formalin and embedded in paraffin. Sections were stained with Gallego’s Trichrome.

### Transfer of MV130-Bacteria From MSCs to DCs

The transfer of MV130-bacteria from MSCs to DCs, generated as described above, was studied by flow cytometry and immunofluorescence. 2 × 10^4^ MSCs were seeded in a 12-well plate and primed with MV130, CFSE-stained MV130 or none (control conditions). After 24 h for allowing the uptake of bacteria by MSCs, cultures were extensively washed and 2 × 10^5^ DCs (1:10 MSC/DC ratio) were seeded. MSCs and DCs were co-cultured for 24 h and the presence of CFSE-MV130 in both populations was analyzed by flow cytometry using CD90 and CD1a, as markers for MSCs and DCs, respectively. For the immunofluorescence study, cells were placed on a chamber slide in a 1:10 ratio (7 × 10^3^ MSCs/7 × 10^4^ DCs). After fixing, cells were stained with phalloidin, HLA-DR and Hoechst as described below.

### Migration Assays

Migration assays were performed in transwell inserts with 8 µm pore membrane (6.5 mm diameter; Costar). After migration, the upper and/or lower fractions were collected and suspended in the same volume. Quantification of cell number was performed by flow cytometry, acquiring all events gated according to forward/side scatter, for 180 s at a constant low flow rate. The percentage of migrated cells were calculated as follow:

(n° of cells present at the lower chamber/total cell number) x 100

To determine if MV130 priming modifies the migratory capability of MSCs, 10^5^ MSCs primed with MV130 (10^6^, 10^7^ o 10^8^ bacteria/mL) or excipient, were seeded in a transwell insert. Cell numbers in each fraction was quantified by flow cytometry after 20 h of culture, as described above.

To assess the possible chemotactic effect of MV130 on MSCs, 10^5^ MSCs were seeded in a transwell insert and placed on a well with culture media with MV130 (10^7^ bacteria/mL), glycerol or IFNγ (10 ng/mL). After 20 h of culture, the number of cells in the upper and lower fractions was quantified by flow cytometry and the percentage of migrating cells were calculated as described above.

To find out if MV130-MSCs have a chemoattractive effect on different leukocyte populations, 2 × 10^5^ MV130-MSCs or CTRL-MSCs were seeded in 24-well flat-bottom culture plates. 10^6^ PBMCs were added into the insert and cultured for 8 h. Migrating cells (present at the lower chamber) were collected and stained for CD14, CD3, CD19, CD56, HLA-DR, and CD90 for flow cytometry analyses. Transwell cultures without MSCs in the lower chamber were used as controls.

### Viability Assays

For cell viability studies, 2 × 10^5^ MSCs were cultured in a 12-well plate. The percentage of apoptotic (Anex^+^/IP^-^) and necrotic (IP^+^) cells was analyzed by flow cytometry after 24 h of treatment, using annexin V conjugated with DY634 (Immunostep) and propidium iodide (Biolegend).

### RNA Extraction and Gene Expression Analysis by qRT-PCR

MSCs were lysed to perform RNA purification using Absolutely RNA Microprep kit (Agilent Technologies) following the manufacturer’s protocol. High capacity cDNA Reverse transcription kit (Applied Biosystems. Thermo Fisher Scientific) was used for the synthesis of the cDNA following the manufacturer’s instructions. Quantitative real-time PCR (qRT-PCR) was performed using specific predesigned TaqMan Gene expression assays for different genes (Applied Biosystems) (**Supplemental Table S1**). All PCR reactions were set in duplicates using the TaqMan Gene Expression Master Mix (Applied Biosystems). The amplification and detection were performed using a 7.900HT Fast Real-time PCR System (Centro de Genómica, Complutense University of Madrid). ΔCT method was employed using *GNB2L1* as reference gene to normalize gene expression.

### Protein Quantification

Production of different cytokines was measured in supernatants from MSC cultures and MSC-Monocyte or MSC-T lymphocyte co-cultures. Levels of TNFα, IL-10 (Biolegend), IFNγ and PGE2 (R&D) were determined by ELISA and levels of IL-6, CXCL8, CCL2, CXCL10 and VEGF-A was determined by Cytometric Bead Array (CBA, BD Bioscience). TGF-β1 production was determined by LegendPlex (Biolegend) according to manufacturer’s instructions.

### Immunofluorescence Analysis

7 × 10^3^ MSCs were grown and treated with MV130 on chamber slides. After fixation with 4% paraformaldehyde and permeabilized with 0.05% saponin, nonspecific epitopes were blocked with PBS containing 10% donkey serum. Then, cells were sequentially incubated with the primary antibody for 45 minutes at room temperature: Texas Red-conjugated-Phalloidin (Thermo Fisher Scientific); anti-CD63 (46); anti-LAMP2 (Developmental Studies Hybridoma Bank at the University of Iowa); anti-HLA-DR (BD Biosciences); anti-paxillin (Sigma Aldrich) as appropriate. Next, cells were incubated for another 45 minutes with the appropriate secondary antibody: Alexa Fluor 594 conjugated donkey anti-mouse IgG; Alexa Fluor 488 conjugated donkey anti-rabbit IgG (both from Invitrogen, Life Technologies); DyLight 405 conjugated goat anti-mouse IgG (Jackson ImmunoResearch). Finally, a counter-staining of the nuclei was performed with Hoechst 33342 (Invitrogen, Life Technologies) for 10 minutes. After staining, preparations were mounted using FluorSave (Millipore) and imaged using a fluorescence microscope (Nikon Eclipse Ci) with a digital camera (Nikon DS-U3) and Nis-Elements D software. Images were assembled using ImageJ software.

### Flow Cytometry

Before staining with specific antibodies, cells were incubated at 4°C for 5 min with FcR Blocking Reagent (Milteny Biotec) to block nonspecific binding. Then, cells were stained with specific monoclonal antibodies (**Supplemental Table S2**) conjugated with different fluorochromes (Alexa Fluor 488, FITC, PE, PerCP, PE-Cy5, Alexa Fluor 647 or APC).

For the intracellular detection of Bcl-2, Bcl-x_L_ and Bax proteins, cells were treated with a FACS permeabilizing solution according to the manufacturer’s instructions (BD Biosciences), and stained with anti-human Bcl-2 or anti-human Bcl-x_L_ Abs (**Supplemental Table S2**) for 30 min. Analysis was performed on a FACSCalibur flow cytometer (BD Biosciences) (Centro de Citometría y Microscopía de Fluorescencia. Complutense University of Madrid) and analyzed with FCS Express V3 software.

### Statistical Analysis

All data are expressed as mean ± SEM of the indicated parameter. Data analysis was performed using GraphPad Prism version 8.0.2. Statistical significance was determined by Wilcoxon test. Values of *p ≤ 0.05, **p ≤ 0.01, and ***p ≤ 0.005 were considered to be statistically significant.

## Results

### Effects of MV130 on MSC Biological Properties

**Figure 1:**
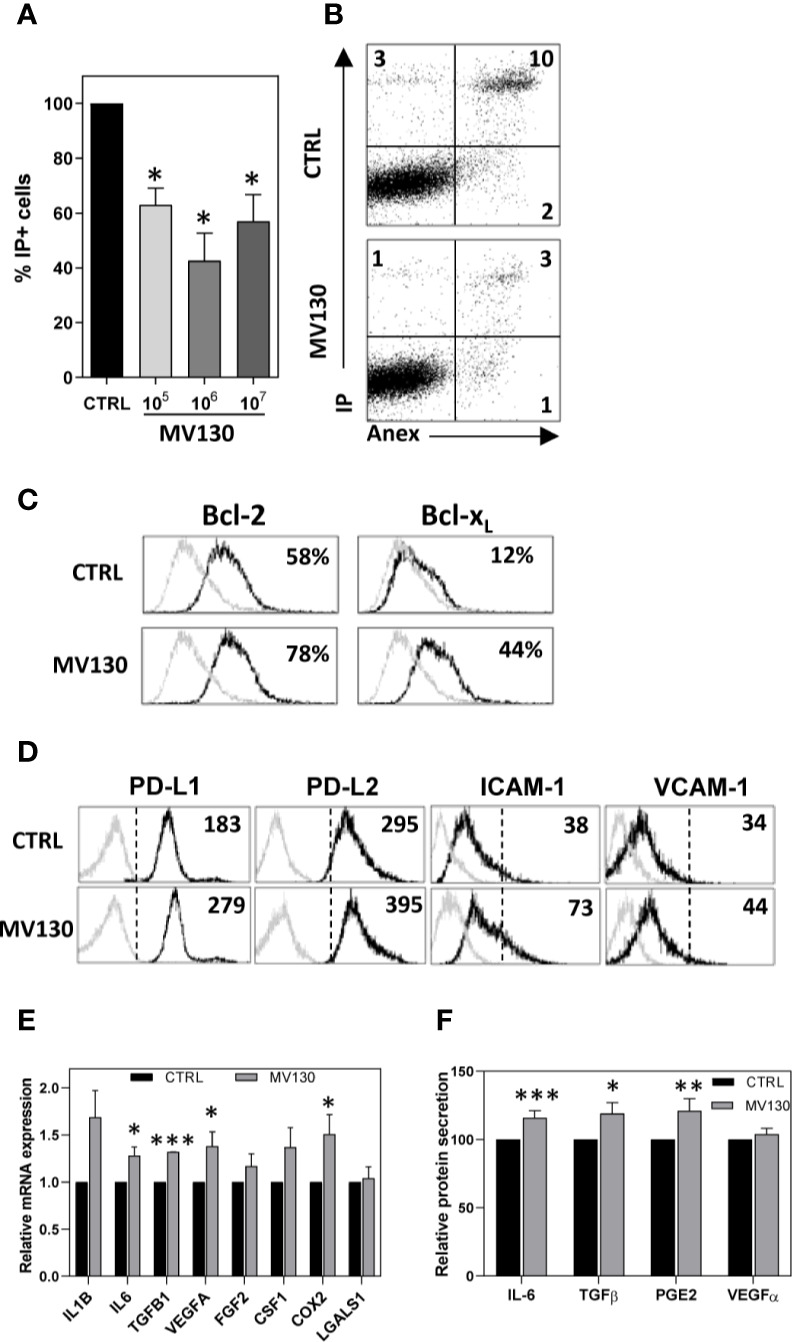
Treatment with MV130 modifies biological properties of MSCs. MSCs were treated with MV130 for 2 h. **(A, B)** After 24 h, MSCs were stained with Annexin V and IP and cell viability was analysed by flow cytometry. Mean ± SEM of 5 independent experiments **(A)** and a representative experiment **(B)** are shown. **(C)** MSCs were treated with MV130 for 2 h. Bcl2 and Bcl-x_L_ expression in MSCs were analyzed by flow cytometry 24 h after treatment. The percentages of positive cells are indicated in each histogram. Gray filled histograms represent isotype control staining. Data are representative of 4 independent experiments. **(D)** 48 h after treatment, the expression of different surface markers was studied on MSCs by flow cytometry. Representative histograms and MFI values are shown (n = 3-4). Gray histograms represent isotype controls. **(E)** mRNA expression for different immunomodulatory factors was studied on MSCs by qRT-PCR. Data represent mean ± SEM of 8 to 12 independent experiments relative to individual controls. **(F)** Supernatants from MSC cultures were collected 48 h after treatment. Protein secretion relative to individual controls is expressed as mean ± SEM from 15 independent experiments (*p < 0.05; **p < 0.01***p < 0.005 by Wilcoxon test).

We first analyzed the effect of MV130 on MSC survival. MV130 significantly increased the viability of MSCs (**Figures 1A, B** and **Supplemental Figure S1A**), increasing significantly Bcl-2 and Bcl-X_L_ antiapoptotic protein expression (**Figure 1C**). **Supplemental Figure S1B** shows that the expression of *NANOG* and *Oct-4* transcription factors, known to be involved in pluripotency and self-renewal of undifferentiated stem cells, were not affected. In addition, no significant differences on the adipogenic and osteogenic differentiation potential were seen when control and MV130-primed MSC cultures were compared (**Supplemental Figures S1C, D**). Thus, while MV130 favors MSC survival, no changes in their stemness and multipotent developmental properties were observed.

MV130 priming of MSCs did not change their expression of *TLR1*, *TLR2*, *TLR3*, *TLR4*, *TLR5*, *TLR6*, and *TLR9* (**Supplemental Figure S1E**), CD86, CD40, or HLA-DR (**Supplemental Figure S1F**) under any of the assayed conditions. CD73, an ectoenzyme with powerful anti-inflammatory properties, was maintained highly expressed in MV130-treated MSCs (**Supplemental Figure S1F**). Relevantly, upon MV130 priming, MSCs significantly upregulated the expression of others immunosuppressive molecules such as PD-L1 and PD-L2, as well as the adhesion protein ICAM-1, key player in MSC-mediated functions (**Figure 1D**). Interestingly, MV130-primed MSCs exhibited enhanced mRNA expression of *IL6, TGF 1, VEGFA* and *COX2* (**Figure 1E**), confirmed at protein level for IL-6, TGF-β1, and for the major COX2 product, PGE2, as detected in MSC culture supernatants (**Figure 1F**).

### MV130-Bacteria Are Uptaken by Oral MSCs *In Vivo* and Reduce Their Migration *In Vitro*

**Figure 2:**
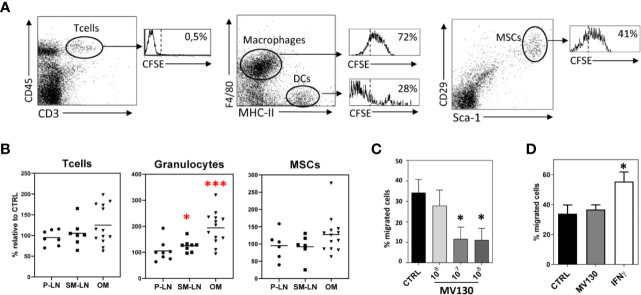
MV130 is uptaken by oral MSCs *in vivo*. **(A)** Flow cytometry analysis of T lymphocytes (CD45^+^CD3^+^), macrophages (F4/80^+^MHC-II^lo^), DCs (F4/80^-^MHC-II^+^) and MSCs (CD29^+^Sca-1^+^) present in the oral mucosa from mice after sublingual immunization with MV130-CFSE. Histograms show the percentage of uptake of CFSE-MV130 by each of these populations. Data are representative of 3 independent experiments. **(B)** Percentage of T cells, granulocytes and MSCs present in peripheral lymph nodes (P-LN), submaxillary lymph nodes (SM-LN) and oral mucosa (OM) from mice after sublingual immunization with MV130 respect to control mice (n = 6–13). **(C)** MV130 priming reduces MSC migration capacity. Bar graph shows the percentage of migrating cells in MV130 primed cultures. Results represent the mean ± SEM of 3 independent experiments **(D)** MV130 does not specifically attract MSCs. Bar graph shows the percentage of migrating cells to MV130 or IFNγ. Results represent the mean ± SEM of 4 independent experiments. (*p < 0.05; ***p < 0.005 by Wilcoxon test).

To determine whether resident MSCs present in the oral mucosa could be responding to MV130 treatment, mice were administered sublingually with MV130-bacteria labeled with CFSE. Flow cytometry analysis of cell suspensions obtained from the excised oral mucosa indicated that MSCs could uptake MV130-bacteria efficiently *in vivo*. As shown in a representative experiment (**Figure 2A**), the percentage of CD45^-^ CD29^+^ Sca1^+^ MSCs uptaking MV130 (41%) was significantly higher to that seen (28%) in MHC-II^+^/F4/80^-^CD19^-^ cells (mostly DCs) but lower than in MHC-II^lo^/F4/80^+^ macrophages (72%). The relative numbers of MSCs and T cells within the oral mucosa remained unchanged upon MV130 treatment, in contrast to the significant recruitment of granulocytes from extramucosal tissues (**Figure 2B** and **Supplemental Figure S2A**).

As no active recruitment of mucosal MSCs was seen in the *in vivo* mouse model after MV130 treatment, we were prompted to explore the migration features of human MSCs primed *in vitro* with MV130. As shown in **Figure 2C**, the migration ability of MV130-primed MSCs was significantly decreased relative to control MSCs. Also, conventional migration assays showed that MV130 did not represent a chemotactic stimulus for MSCs, unlike IFNγ used as positive control (**Figure 2D**). In consonance with the migration assays, the immunofluorescence study revealed that MV130-primed MSCs showed characteristics of slow-moving cells with sparse and large streak-like focal adhesion complexes at the periphery and a low polymerization degree of F-actin, whereas control MSCs showed small dot-like nascent focal adhesion sites and polymerized actin at the ruffles (**Supplemental Figure 2B**). Thus, the results indicate that MSCs may uptake MV30-bacteria while decreasing their migratory activity.

### MSCs Act as Reservoirs of MV130 and Are Able to Transfer it to DCs

**Figure 3:**
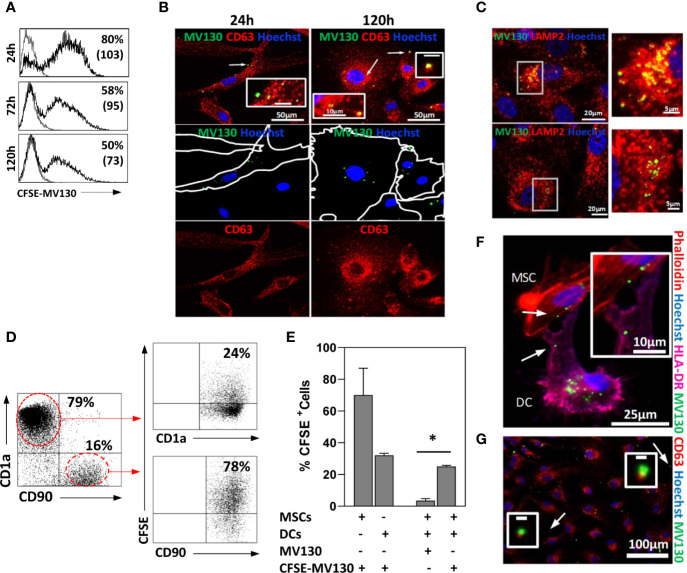
MSCs act as reservoirs of MV130 and are able to transfer it to DCs. MSCs were treated for 24 h with MV130 labeled with CFSE (CFSE-MV130; green) for monitoring. After washing cells to remove the drug, its uptake, processing and transference to DCs were studied. **(A)** Uptake and maintenance of CFSE-MV130. Histograms show the percentage of positive cells and MFI in brackets (n = 6–8 independent experiments). **(B, C)** Spatiotemporal monitoring of MV130 (green) in MSCs. Immunostaining in red for CD63 **(B)** or LAMP2 **(C)** in MV130-MSCs at different times. Inset indicated by arrows in B, scale bar: 10μm (24 h) or 1μm (120 h). Right: Higher magnification of the white square in **(C)** (120 h); examples of LAMP2-positive compartments with CFSE-MV130. Hoechst was used for nucleus staining (blue). Images are representative of 5 independent experiments. **(D–G)** DCs differentiated from monocytes were co-cultured with MSCs, previously treated with MV130 or CFSE-MV130 as described in Material & Methods section, and transfer of MV130 from MSCs to DCs was studied. A representative dot plot **(D)** and the mean ± SEM of five independent experiments **(E)** are shown. DCs and MSCs were gated according to CD1a or CD90 expression, respectively. In **(E)** control MSCs and DCs directly treated with CFSE-MV130 are also shown. **(F)** MV130 transfer from MSCs to DCs studied by immunofluorescence. Hoechst was used for nucleus staining in all cases (blue). Co-cultures were labeled with phalloidin (red) and anti-HLA-DR (magenta). The absence of HLA-DR expression on MSCs allow to distinguish it from DCs (HLA-DR^+^). White arrows in **(F)** indicate area of insert image magnification. **(G)** MSCs were labeled with anti-CD63 (red) and Hoechst was used for nucleus staining (blue). Co-localization of CD63^+^ extracellular microvesicles with CFSE-MV130 was observed free in the medium. White arrows indicate area of insert image magnification. Representative images of 3 independent experiments (*p < 0.05 significance by Wilcoxon test).

Next, we studied the fate of MV130-bacteria uptaken by MSCs. To this end, cells were treated 24 h with CFSE-labeled MV130 and then extensively washed to remove extracellular bacteria. Bacteria-containing MSCs were monitored by flow cytometry and studied by immunofluorescence at different times. After treatment, about 60-85% of MSCs showed fluorescent staining. Although this percentage gradually decreased in the following days, around 50% of the MSCs still showed significant levels of fluorescence 5 days later (**Figure 3A**). As shown under microscopy (**Figure 3B**), MV130-bacteria accumulated in the vesicular compartment near the plasma membrane at the beginning, suggesting their internalization into early endosomes. Later (120 h) they appeared translocated to the late endosomal/lysosomal compartments as indicated by the co-localization with CD63 and to a lesser extent with LAMP2 markers (**Figures 3B, C**). These results indicate that MSCs are able to internalize, process and maintain the MV130-bacteria over time.

As it has been shown that MSCs may retain TLR2 ligands and transfer them to immune cells (47), we were prompted if this might be also the case with MV130-bacteria. Thus, MSCs were treated with CFSE-labeled MV130, extensively washed to remove unbound bacteria and co-cultured with monocyte-derived DCs. After 24 h, a notable proportion (around 25%) of DCs co-cultured with MSCs showed significant labelling with CFSE-conjugated MV130, compared to only 2% of background staining (**Figures 3D, E**). Immunofluorescence studies of MSC-DC co-cultures showed that CFSE-labeled bacteria could be transferred from MSCs to neighboring DCs through transient cytoplasmic extensions (**Figures 3F**). In addition, CFSE-MV130 conjugates from MSCs could also reach DCs through extracellular vesicles since in the co-cultures it was possible to observe CD63^+^ vesicles loaded with CFSE-conjugated MV130 (**Figure 3G**). Interestingly, in control experiments using only DCs, a direct uptake of CFSE-MV130 by these cells was around 35%, whereas it reached to around 70% when only MSCs were used (**Figure 3E**), which underlined the high ability of MSCs to uptake MV130-bacteria.

### Priming MSCs With MV130 Promotes Their Immunomodulatory Features Enhancing the Induction of M2-Like Macrophages

**Figure 4:**
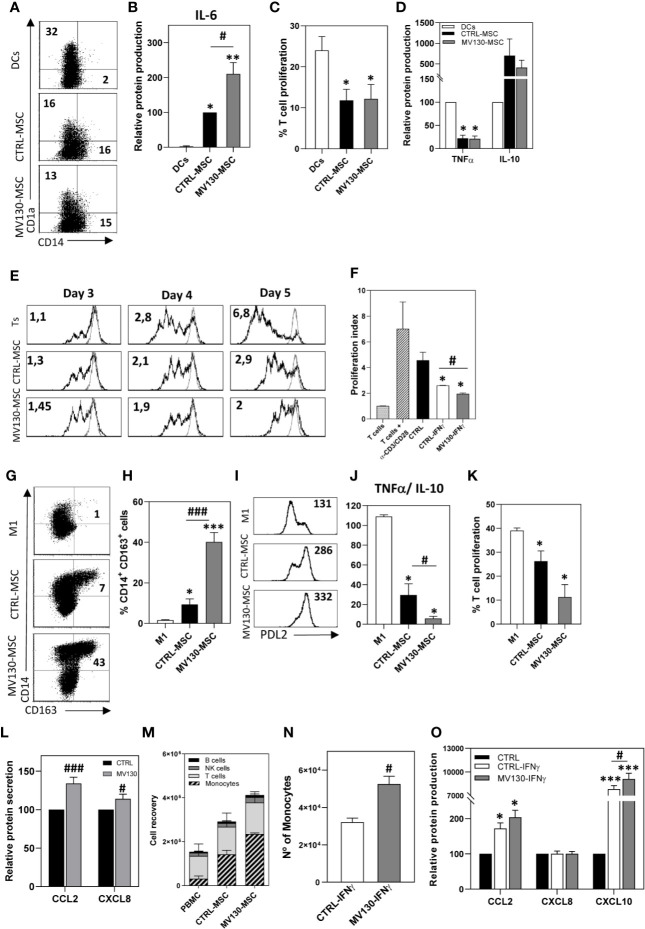
Immunomodulatory abilities of MSCs after activation with MV130. **(A–D)** Phenotype and function of monocyte-derived DCs differentiated in the presence or absence of CTRL-MSCs or MV130-MSCs. At day 6, CD1a and CD14 expression were analyzed by flow cytometry in the CD90^-^ population. The percentage of positive cells is shown in each plot **(A)** and IL-6 production was measured in the supernatants **(B)**. Results represent the mean ± SEM (n = 6). **(C, D)** DCs stimulated with LPS were cultured in MLR assays with CFSE-labeled T lymphocytes. After 5 days, the percentage of proliferating T cells was calculated by CFSE dilution method (gated on CD3^+^ cell population) **(C)** and supernatants from MLR co-cultures were analyzed for TNFα and IL-10 protein secretion **(D)**. Data represent the mean ± SEM (n = 3–5). **(E)** Control or MV130-MSCs were co-cultured with CFSE-labeled T lymphocytes stimulated with CD3/CD28 beads, for different times. Histograms show CFSE staining in proliferating T cells in CD3^+^ gated cells. Proliferation index referred to unstimulated T lymphocytes (gray line) is indicated. Data are representative from four independent experiments. **(F)** MV130-MSCs re-stimulated with IFNγ, following protocol described in Material and Methods, were co-cultured with CFSE labeled T lymphocytes. Proliferation index is shown. Bar graph shows mean ± SEM (n = 4). **(G–K)** Control and MV130 primed MSCs were co-cultured with monocytes in the presence of GM-CSF to induce M1 macrophage differentiation. Monocytes alone were cultured as M1 control. **(G–I)** After 6 days, CD14, CD163 and PD-L2 expression was determined by flow cytometry in non-MSC population (CD90^-^ cells) (n = 5–6). A representative experiment **(G)** and mean ± SEM of percentage of CD14^+^CD163^+^CD90^-^ cells from five to six independent experiments **(H)** are shown. **(I)** Representative PD-L2 expression on macrophages. MFI is shown in each histogram. **(J)** After 6 days of co-culture, LPS was added and supernatants were analysed for TNFα and IL-10 production. Data represent TNFα/IL-10 ratio production at the different experimental conditions (mean ± SEM; n = 4). **(K)** Macrophages stimulated with LPS were used to carry out MLR cultures with CFSE-labeled T lymphocytes. After 5 days, the percentage of T cell proliferation was measured in the CD3^+^ cell population. **(L)** CCL2 and CXCL8 protein secretion measured in control and MV130-MSC culture supernatants. Bars represent the mean ± SEM relative to individual controls from 15 independent experiments. **(M)** PBMCs were placed in a transwell insert while MSCs, treated with or without MV130 for the 24 h previous, and seeded in the bottom chamber. After 8 h, migrating PBMCs (present in the lower chamber) were collected and stained for CD14, CD56, CD3, HLA-DR, and CD19, and different leukocyte populations were analyzed by flow cytometry. MSCs were excluded from the analysis by CD90 expression. (mean ± SEM; n = 4) **(N)** PBMCs migrating toward control or MV130 primed MSCs re-stimulated with IFNγ. Monocyte recruitment was analyzed by flow cytometry (mean ± SEM, n = 4). **(O)** Supernatants from MSC cultures following the protocol described in Material & Methods section were analyzed for CCL2, CXCL8, and CXCL10 protein secretion after IFNγ re-stimulation. Results represent mean ± SEM of four to six independent experiments relative to individual controls. (*p < 0.05; **p < 0.01, ***p < 0.005 significances relative to M1-macrophages or DC; ^#^p < 0.05; ^###^p < 0.005 significances relative to CTRL-MSCs by Wilcoxon test).

Both control and MV130-primed MSCs similarly dampened the generation of CD14^–^ CD1a^+^ DCs from CD14^+^ CD1a^–^ monocytes (**Figure 4A**). DCs induced in the presence of MV130-primed MSCs showed a slight but constant increase in the expression of HLA-DR, PD-L1 and PD-L2, while decreasing CD86 co-stimulatory molecule expression (**Supplemental Figure S3A**) and inducing a significant increase of IL-6 production (**Figure 4B**). These DCs showed a similarly reduced allostimulation ability in co-cultures with T cells (**Figures 4C, D**).

To assess the effects of the priming of MSCs with MV130 on the adaptive immune response, the ability of MSCs to modulate TCR-triggered activation of T cells was also investigated by proliferation assays. As shown in **Figure 4E**, in the presence of MSCs, T cell proliferation was slightly increased on day 3 while significantly suppressed on day 5. These enhancing and suppressing effects were more evident when MV130-primed MSCs were used, reaching signification on day 5 (**Figure 4E** and **Supplemental Figure S3B**). On the other hand, to test whether this response was transient or could be maintained for a prolonged period of time, MSCs were primed with MV130 for 24 h, extensively washed to remove extracellular bacteria and then stimulated with IFNγ on day 4. **Figure 4F** shows that CD3/CD28-induced T-cell proliferation was inhibited by IFNγ-activated MSCs in a greater extent than non-activated MSCs; yet, such an inhibition was significantly higher using MSCs primed with MV130.

We also addressed whether MV130 priming could influence the effects of MSCs on the repolarization of monocytes toward M2-like macrophages. As shown in **Figures 4G–I**, monocytes undergoing differentiation to macrophages in the presence of MSCs showed a M2-like pattern. This effect was more notable in cultures with MV130-primed MSCs, where the proportion of CD14^high^ CD163^high^ PD-L2^+^ macrophages generated was significantly increased respect to control MSC cultures (**Figures 4G–I**). After 6 days of differentiation, macrophages generated in the different cultures were activated with LPS and the cytokine production was analyzed. As shown in **Figure 4J**, macrophages differentiated in the presence of MSCs showed a reduced pro-inflammatory TNFα/anti-inflammatory IL-10 cytokine production ratio when compared with control M1-like macrophages, and this reduction was more pronounced in macrophages generated in the presence of MV130-primed MSCs with significantly increased IL-10 levels (p<0.05). In addition, macrophages generated in the presence of MV130-MSCs induced a lower proliferative response of T cells than their control counterparts (**Figure 4K**). As recent studies have demonstrated that MSC-derived CCL2 is required for polarizing IL-10^+^ tissue macrophages (48–50), the effect of priming MSCs with MV130 on its production was studied. As shown in **Figure 4L**, CCL2 production was upregulated in MV130-primed MSCs at both mRNA and protein levels (**Supplemental Figure S3C**). This was also the case for CXCL8 and CXCL12 (**Figures 4L** and **Supplemental Figure S3C**). Since CCL2, CXCL12 and CXCL8 are the major chemokines driving monocyte extravasation, chemotaxis assays were performed with PBMCs in a transwell system. The results showed that monocytes were the main cell type migrating at higher numbers toward the compartment with MV130-primed MSCs, as compared with untreated MSCs (**Figure 4M** and **Supplemental Figure S3D**). In addition, priming of MSCs with MV130 also modified their capacity to recruit monocytes upon IFNγ re-stimulation (**Figure 4N**). Again, this effect was accompanied by an increase in CCL2, and also CXCL10, chemokine production (**Figure 4O**).

### Effects of MV130-Primed MSCs in an *In Vivo* Model of Acute Inflammation

**Figure 5:**
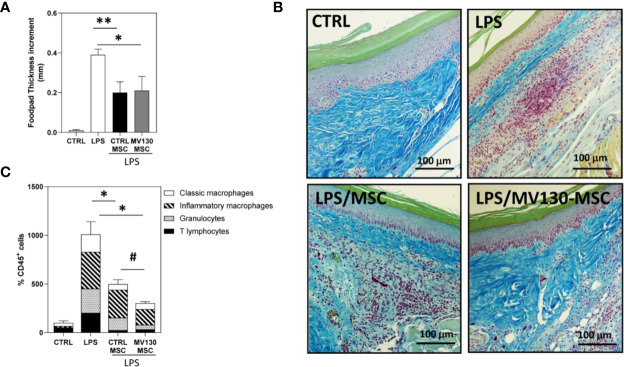
Effects of MV130-MSCs in an *in vivo* model of acute inflammation. FVB/NJ mice were challenged in the footpad with 40μg of LPS and administered with or without control or MV130-primed MSCs 24 h later. **(A)** Footpad thickness increment was determined after 72 h as a measure of the efficacy of the different experimental groups of MSCs. Data shown are mean ± SEM of two independent experiments (three mice per group) **(B)** Images show histological sections of footpad tissue stained with Gallego’s Trichrome. Images are representative of 3 mice per group. **(C)** Percentage of CD45^+^ leukocytes infiltrating footpads in the different mouse groups analyzed by flow cytometry. The distribution of the different leukocyte subpopulations in CD45^+^ cells is also shown in each experimental group. Results represent increments relative to control animals (2 independent experiments with 3 mice per group) (*p < 0.05,**p < 0.01 versus LPS alone; ^#^p < 0.05 versus CTRL-MSCs; by Wilcoxon test).

As both MSCs and M2-like macrophages are associated with wound healing and tissue repair, we were prompted to test whether MV130-primed MSCs may have a differential effect in an *in vivo* model of acute inflammation. To this end, mice were injected in the footpad with LPS alone or with control or MV130-primed MSCs 24 h later. Both MSCs significantly reduced the LPS-inflammatory response, measured by footpad thickness, without differences between MV130-primed and control MSCs (**Figure 5A**). However, the histological analysis showed a clear reduction of leukocyte infiltration in those mice co-injected with MSCs primed with MV130 (**Figure 5B**). Flow cytometry analysis of the excised tissues indicated that leukocyte infiltration was markedly decreased by the treatment with MV130-primed MSCs, in comparison to that using control MSCs (**Figure 5C**). As shown, granulocytes and inflammatory macrophages were reduced by 50–60% and 40–50%, respectively (**Figure 5C**).

## Discussion

The oral mucosa constitutes an essential body barrier constantly exposed to both potentially harmful and harmless antigens. Histologically consists of a stratified squamous epithelium and a connective tissue, the lamina propria, where specialized immune cells function providing protection from pathogens and tolerating commensal microorganisms (51). However, in the last years some studies have pointed out the relevance of oral mucosal non-immune cells, such as MSCs, in the maintenance of tissue homeostasis and also in the immune reactions, producing anti-microbial factors and finely regulating the response to pathogens (20, 26, 27).

Numerous groups have reported that MSCs are equipped with a large variety of TLRs capable to act as sensors of exogenous stimuli, and also endogenous signals related to tissue damage or inflammation (33). In addition, Iwamura et al. have recently demonstrated the NOD1 expression in bone marrow MSCs (52). This arsenal of PRRs may then be essential for MSC recognition of the PAMP ligands from the polibacterial preparation used in the present study, similar to that described for dendritic cells (17). Although it has been previously shown that the TLR expression pattern could be modulated by several environmental conditions, including hypoxia or inflammation (33), our mRNA expression study reveals that MV130 treatment does not modify TLR expression in MSCs, at least in basal conditions. There is currently no consensus on whether TLR activation can affect MSC differentiation capacities, what seems to depend on the type of TLR ligand (53), but an important issue in the context of vaccination with MV130 is the fact that MSCs primed with MV130-bacteria maintain their stemness and multilineage potential. Also, MV130 primed MSCs maintain low immunogenicity due to the lack of HLA class II and co-stimulatory molecule expression after treatment, and in agreement several authors have described that the activation of different TLRs has no significant effect on the immunogenic properties of MSCs (53, 54).

An important proportion of MSCs found in the lamina propria of the oral mucosa contains the polybacterial preparation after sublingual immunization of mice with MV130. How the bacterial preparation reaches MSCs located in the mucosa connective tissue is unknown, but DCs infiltrating the epithelium could be involved in the transfer of bacterial components to MSCs (data not shown) (55). Previous work focused on the distribution and effectiveness of DCs after sublingual vaccination with cholera toxin showed that MHC class II^+^ cells were quickly recruited to the sublingual mucosa, where they processed the antigens and immediately transported them *via* afferent lymph to draining lymph nodes (56). Unlike DC behavior, our *in vivo* results point out that, after MV130 administration, oral-resident MSCs remain in the mucosa, in correlation with the *in vitro* assays showing inhibition of MSC migratory abilities after MV130 treatment. To this respect, it has been demonstrated that stimulation of some TLRs could inhibit MSC migration, thus facilitating their local action (57, 58). Our results show that MSCs, apart from capturing the components of the polybacterial preparation, can process and retain a reservoir of the TLR ligands derived from MV130 digestion. Previous work has described the capacity of MSCs to uptake and process proteins although, unlike antigen presenting cells, they do not stimulate directly alloreactive T cells (59). Additionally, Weinstock et al. showed that MSCs were able to retain, in a long-term manner, TLR2 ligands which were subsequently released and transferred to immune cells, inducing a pro-inflammatory response (47). Our data also indicate that MSCs can transfer processed MV130 components to DCs either directly, through cell-to-cell contacts, or indirectly through extracellular vesicles. This fact could contribute significantly to the described beneficial effects of MV130 in the control of recurrent infections, since DCs could be activated more quickly and by a lesser amount of TLR ligands in subsequent infections.

The immunoregulatory capacity of MSCs is largely governed by the local inflammatory intensity, being able to promote an inflammatory response or, conversely, prevent an excessive immunoreaction (60). A complex balance among opposite stimuli guide this striking functional plasticity of MSCs, allowing to adapt MSC responses according to time and course of infection (28). Based on these properties, previous studies have shown that different priming approaches to empower MSCs, including pro-inflammatory cytokines, hypoxia or TLR ligands, modify their immunophenotypic and secretome profiles (61), and as consequence their immunoregulatory effects. In this context, our results show that MV130 treatment of MSCs maintains such plasticity improving each of the immunoregulatory faces of MSCs. Priming with MV130 seems to increase the previously described pro-inflammatory activities of MSCs during early-stage inflammation, initially favoring leukocyte recruitment, primarily monocytes and granulocytes, through the enhanced production of chemokines, as described by Waterman et al. after TLR4 triggering (31). The upregulated expression of adhesion molecules, such as ICAM-1, observed in MV130-primed MSCs could also facilitate close interactions between MSCs and leukocytes, including T cells. MV130 priming of MSCs initially leads to an increased T-cell activation, presumably as a consequence of the higher production of pro-inflammatory cytokines and chemokines (IL-6, CXCL8 and CCL2). TLR4 activation in MSCs has been described to produce similar effects to these reported in our study (31, 62). As proposed by the *licensing* model, the expression levels of inflammatory cytokines, including IFNγ produced by T-cells, determine the immunosuppressive activity of MSCs as shown in different preclinical models (50, 63, 64). In our study, the threshold levels controlling the change in MSC behavior would be reached in the MV130-primed MSC/T cell co-cultures faster than in the control cultures, causing the greater inhibitory effect observed on T cell proliferation. Our data also indicate that MV130-primed MSCs are able to respond more intensively when are subsequently exposed to an inflammatory microenvironment, inhibiting T cell activation. As Liu and colleagues showed, MSCs exhibit short-term memory when are exposed for a second time to danger signals, suggesting that trained immunity could be also carried out by these non-professional immune cells (65). As described for other cells (14), MV130 treatment imprint an innate immune memory-like in MSCs allowing them to give a better response to a subsequent damage stimulus.

On the other hand, we found that the priming of MSCs with MV130 induces a higher expression of IL-6, a known negative regulator of DC differentiation and function (66, 67). This could explain the changes observed in the phenotype of DCs generated in the presence of MV130-primed MSCs, exhibiting higher levels of PD-L1 and PD-L2 inhibitory molecules. However, these DCs generated in the presence of MV130-treated MSCs show a drastically reduced allostimulatory capacity, very similar to those DCs cultured with control MSCs.

Different preclinical models have described a pivotal role for tissue macrophages as part of the therapeutic response to MSCs, contributing to their anti-inflammatory effects (38, 50, 68). An important result of the present study is the impact of MV130 priming of MSCs in macrophage polarization and function. MV130-primed MSCs altered monocyte differentiation during M1-like polarization, inducing highly immunosuppressive M2-like macrophages associated with reduced TNFα and increased IL-10 production together with a higher expression of PD-L2 inhibitory molecule. Similar to that previously discussed for T cell activation, the production of pro-inflammatory cytokines by monocytes during their M1-like polarization would constitute a stronger activation stimulus for MV130-primed MSCs than for control MSCs, triggering the release of mediators that skew the differentiation of monocytes toward a more anti-inflammatory profile. Mechanistically, the activation of the COX2*/*PGE2 pathway, as well as the increase of both PD-L2 expression and mainly CCL2 production in MV130-primed MSC cultures, could favor the promoting effects on M2 polarization, as has been described in other systems (69, 70). *In vivo* and *in vitro* studies have demonstrated that MSC-derived CCL2, apart from its role in chemotaxis, has a pivotal immunosuppressive function and is required to polarize macrophages toward an IL-10^+^ M2-like phenotype (48–50). MSC-derived MMPs are able to proteolytically process CCL2 to generate an N-terminal-cleaved form with anti-inflammatory functionality (48). Alternatively, CCR2 ligands may heterodimerize and form oligomers which induce MCP-induced proteins (MCPIP1-4), able to specifically promote IL-6 mRNA decay and upregulate M2-associated *c-Maf* gene favoring M2-like macrophage polarization (50, 71, 72). Thus, it could be hypothesized that priming MSCs with MV130 would be advantageous for the resolution of inflammation, also through the enhanced monocyte recruitment and subsequent differentiation to IL-10^+^ anti-inflammatory macrophages. In this sense, in the *in vivo* LPS acute inflammation model used in the current study, the anti-inflammatory effects were accentuated when MSCs had been pretreated with MV130 reducing drastically leukocyte infiltration, mainly inflammatory macrophages and granulocytes. These results suggest that MV130 priming allows MSCs to reach their activation threshold more quickly and thus exert their anti-inflammatory effects more efficiently, limiting macrophage activation to avoid excessive tissue damage.

## Conclusion

**Figure 6:**
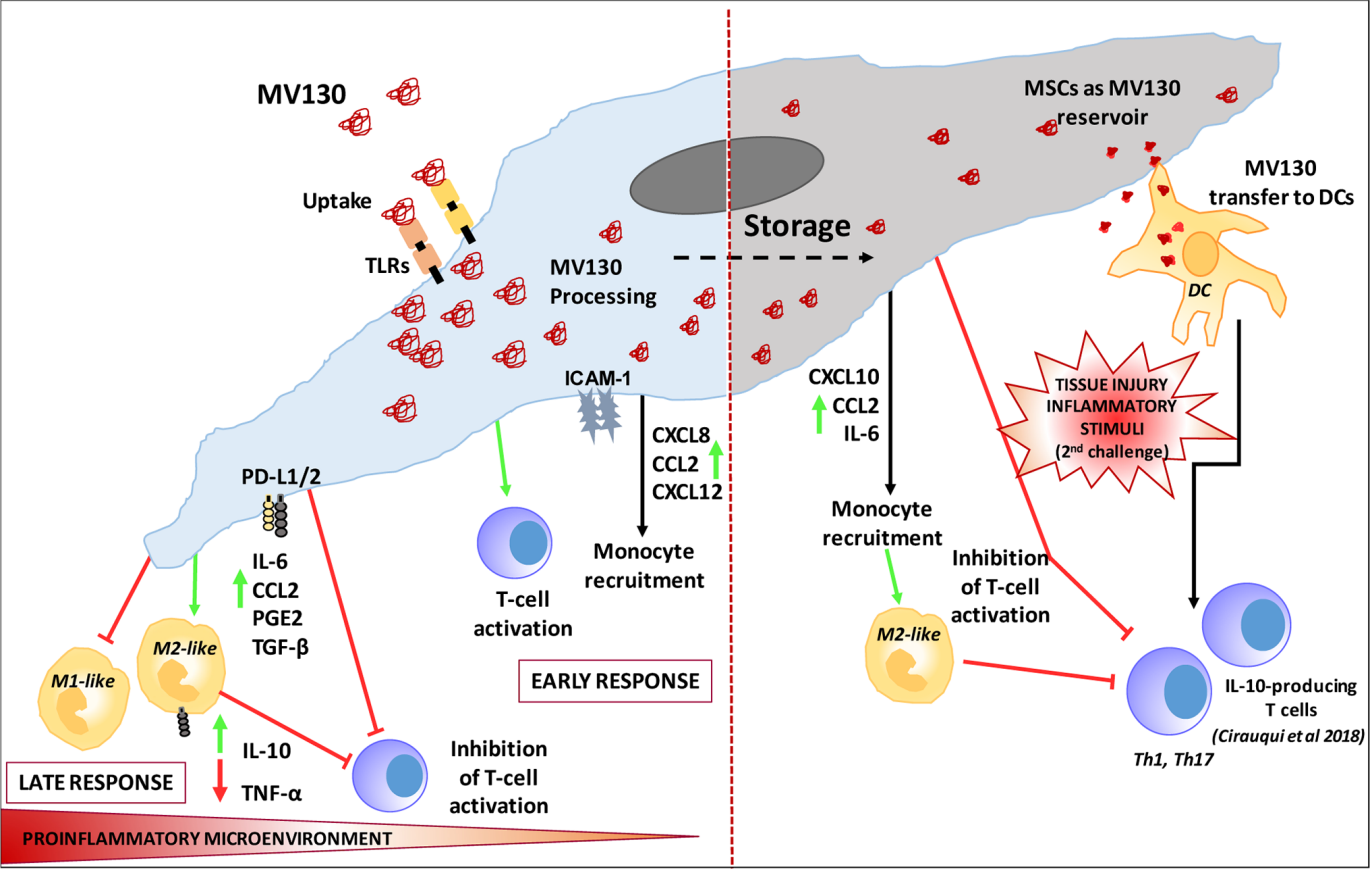
Proposed Model of MSC involvement in oral mucosal bacterial immunotherapy with MV130.

Resident oral mucosa MSCs are able to uptake, process and retain a reservoir of the TLR ligands derived from MV130 digestion. MV130 treatment of MSCs improves each of the immunoregulatory faces of MSCs. Initially during early-stage inflammation, MV130-primed MSCs favors leukocyte recruitment and T-cell activation, through the enhanced production of chemokines. Once exposed to sufficient levels of pro-inflammatory cytokines, MV130-primed MSCs respond by adopting an improved immune-suppressive phenotype, to dampen inflammation and avoid excessive tissue damage, reducing T lymphocyte proliferation, and mainly favoring IL-10^+^ M2-like macrophage differentiation through upregulation of immune-supressor factors (CCL2, PGE2, TGF-β, and PD-L1/2). MV130-primed MSCs show additional features that can be associated to trained immunity since they modify their response to a secondary inflammatory stimulation, inhibiting more efficiently T cell activation and producing higher levels of CXCL10 and CCL2 chemokines. Furthermore, MSCs can transfer processed MV130 components to DCs allowing them to be activated more quickly and by a lesser amount of TLR ligands in an inflammatory microenvironment. Therefore, we propose that MSCs could be involved in the observed clinical benefits of MV130 (**Figure 6**).

## Data Availability Statement

The raw data supporting the conclusions of this article will be made available by the authors, without undue reservation.

## Ethics Statement

All experimental procedures were carried out according to Spanish and European regulations (Spanish RD 53/2013 and Law 6/2013, European Directive 2010/63/UE). Procedures were approved by the CIEMAT Animal Experimentation Ethical Committee according to approved biosafety and bioethics guidelines.

## Author Contributions

AVá performed research, experimental animal model, data analysis, and wrote manuscript. LF-S performed research. EJ, DP-C and RY performed experimental animal model. JS final writing and editing. AVa designed research, performed data analysis and final writing and editing. JV designed research, supervised the study, performed research, data analysis, and wrote the manuscript. AVi designed research, supervised the study, performed data analysis, and wrote the manuscript. All authors contributed to the article and approved the submitted version.

## Funding

This work was supported by grants, INMUNOTEK, S.L. (105-2017-A-2020); RTI2018-093899-B-I00 (Spanish Ministry of Economy and Competitiveness), RD16/0011/0002 (Institute of Health Carlos III, Spain), and B2017/BMD-3692 AvanCell-CM (Community of Madrid).

## Conflict of Interest

JS is the CEO of Inmunotek SL, a pharmaceutical company that manufactures bacterial vaccines. The authors declare that this study received funding from Inmunotek SL. The funder had the following involvement in the study: final writing and editing of the manuscript.
