# Supplementary material for: Involvement of Mesenchymal Stem Cells in Oral Mucosal Bacterial Immunotherapy
Source: Front Immunol. 2020 Nov 19;11:567391. doi: 10.3389/fimmu.2020.567391 (PMC7711618; doi:10.3389/fimmu.2020.567391)
Supplement: Supplementary file 1 [file DataSheet_1.pdf]

SUPPLEMENTAL FIGURE S1

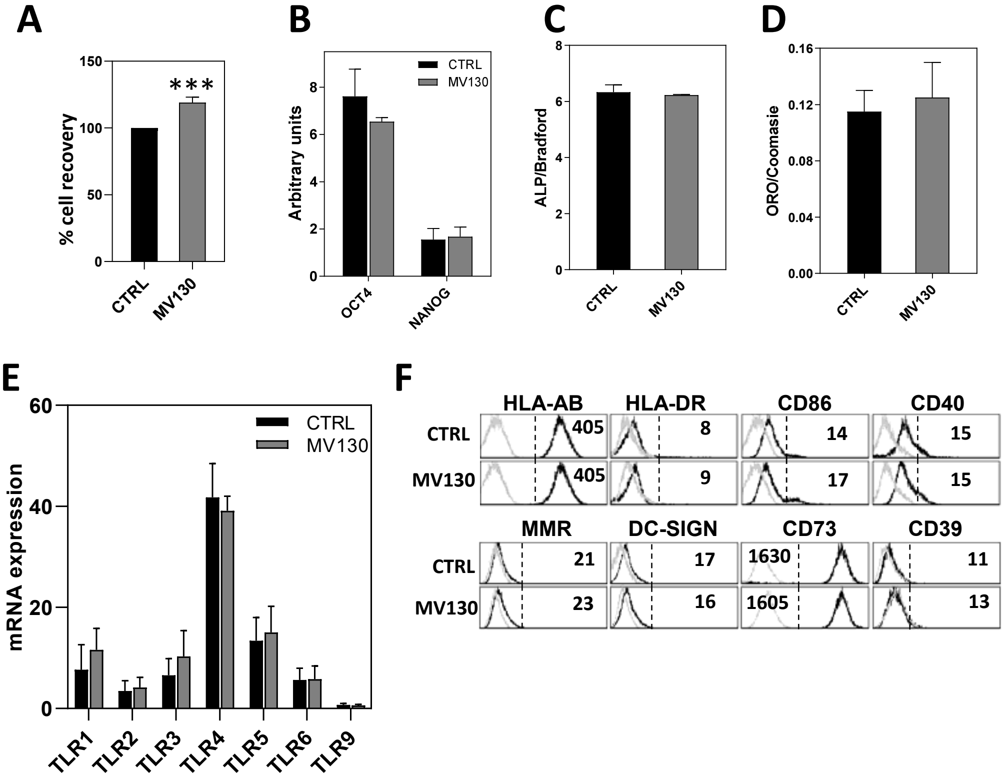

(A) Number of MSCs recovered from control or MV130 treated cultures after 24h. Data represent the percentage of MV130 treated cultures relative to control ones expressed as mean ± SEM from 15 independent experiments**.** **(B)** mRNA expression of Oct-4 and NANOG in MV130-treated MSCs. Mean ± SEM is represented (n=3). **(C-D)** MSCs were treated with or without MV130 as described in material & methods section, before undergoing adipogenic or osteogenic differentiation process. For osteogenic **(C)** and adipogenic **(D)** differentiation, ALP specific activity /protein concentration ratio and lipid content expressed as ORO/Coomasie blue stain ratio respectively, were measured (n=2 independent experiments). **(E)** mRNA expression of different TLRs in control and MV130-MSCs after 2h of treatment. Bars graph represent mean ± SEM of three independent experiments. **(F)** 48h after treatment, the expression of different surface markers on MSCs was studied by flow cytometry. Gray histograms represent isotype controls. Representative histograms and MFI value are shown (n=3-4).

SUPPLEMENTAL FIGURE S2

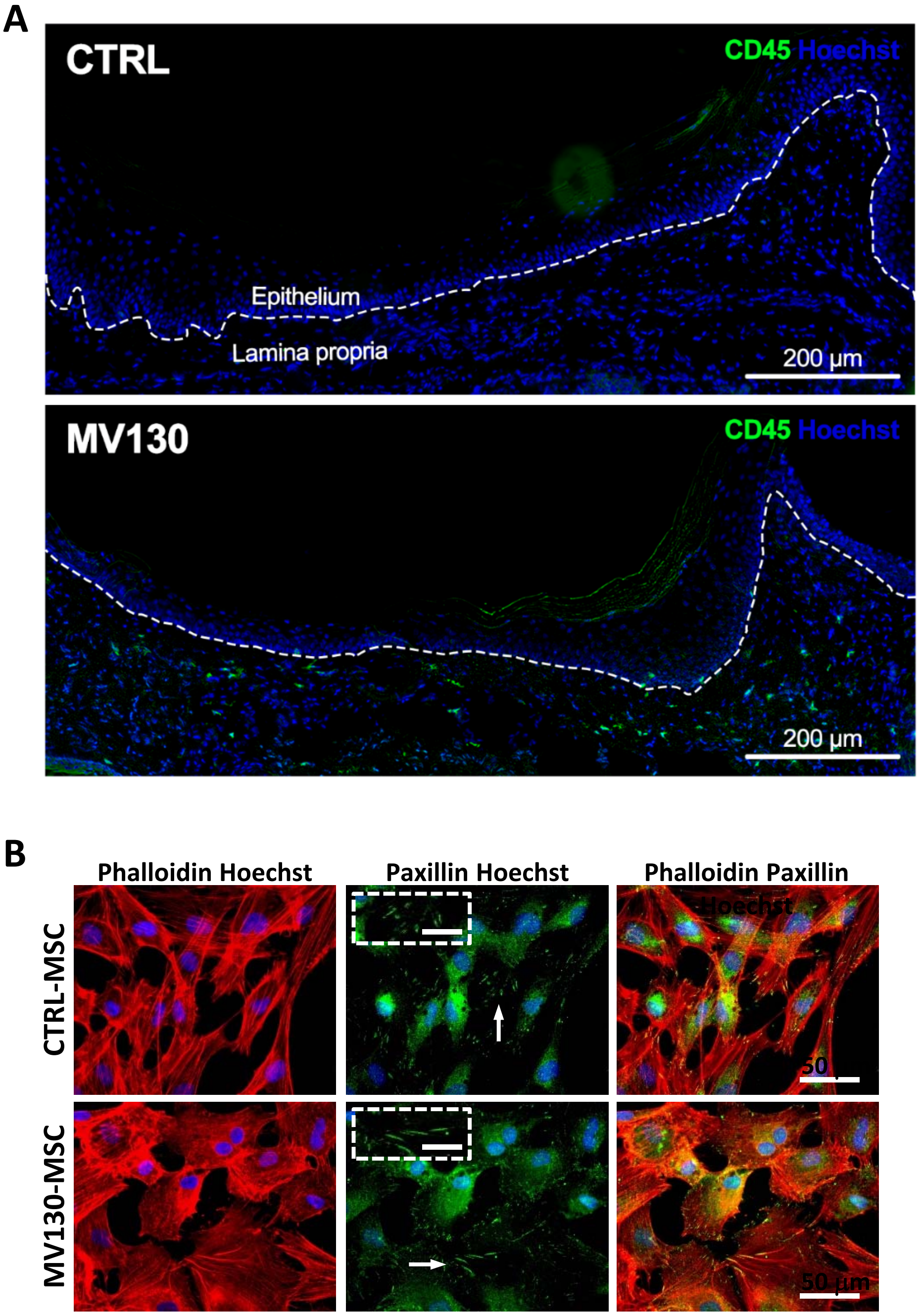

(A) Recruitment of CD45^{+} cells in the oral mucosa of mice after sublingual vaccination with MV130. Images show histological sections of oral mucosa stained with anti-CD45 (green). Hoechst was used for nucleus staining (blue). Images are representative of 2 mice per group. **(B)** Effect of MV130 on paxillin expression in MSCs. MV130-treated MSCs were stained for F-actin (phalloidin, red) and paxillin (green). Hoechst was used for nucleus staining (blue). White arrows indicate area of insert image (green channel at higher magnification, scale bar: 25 µm). Data are representative of 3 independent experiments.

SUPPLEMENTAL FIGURE S3

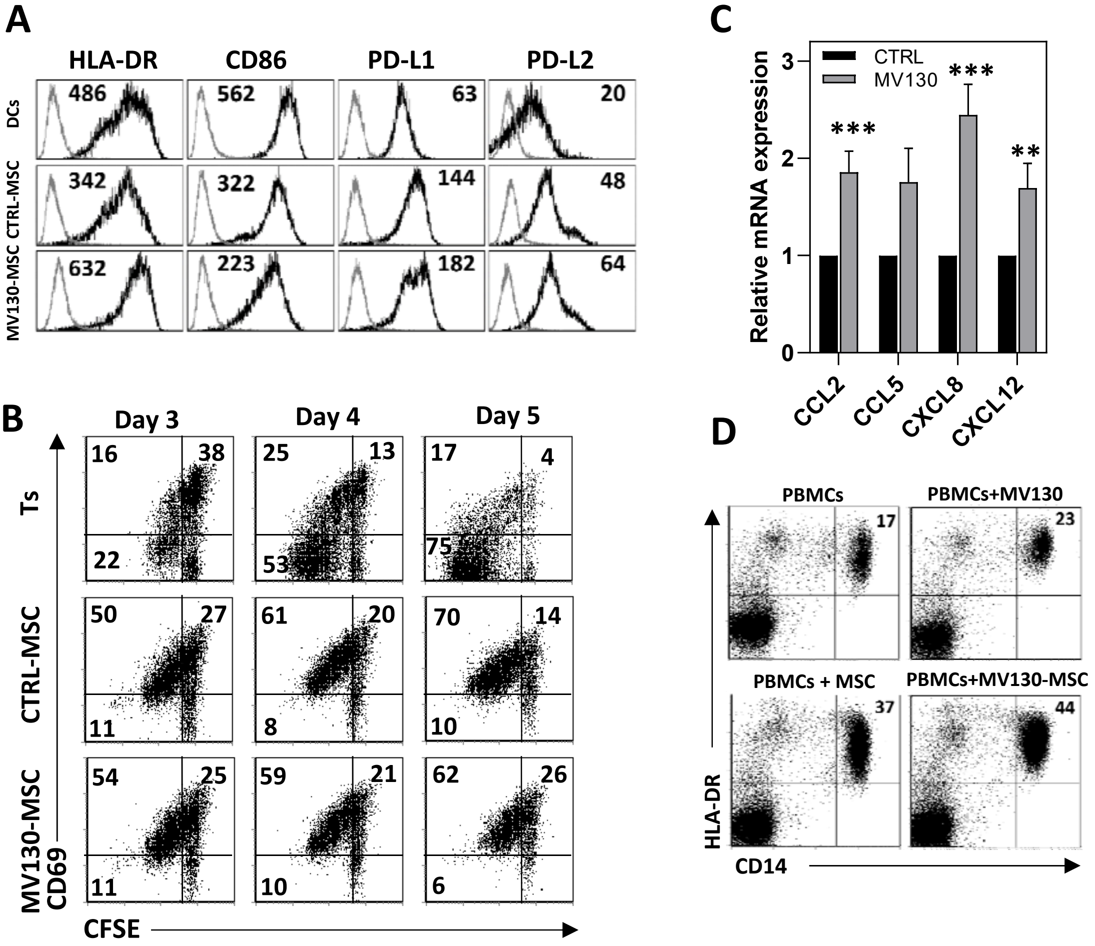

(A) Control and MV130 primed MSCs were cocultured with monocytes in the presence of GMCSF+IL-4 to induce DC differentiation. At day 6, the expression of different surface markers was analyzed by flow cytometry (black), in $CD90^-$ population, compared with background fluorescence (light gray). MFI is shown in each histogram. Data are representative of five independent experiments. **(B)** Control or MV130-MSCs were cocultured with CFSE labelling T lymphocytes, stimulated with CD3/CD28 beads, for 3, 4 an d5 d ay s Stimulated T cells alone were carried out as control. Percentage of $CFSE^+CD69^+$, $CFSE^-CD69^+$ and $CFSE^-CD69^-$ populations are shown in each plot. Data are representative of four independent experiments. **(C)** mRNA expression for different chemokines in control and MV130-MSCs after 2h of treatment. Data represent mean ± SEM respect to control cultures of 8 to 12 independent experiments. **(D)** PBMCs were placed in a transwell insert while control or MV130-MSCs were seeded in the bottom chamber. After 8h of migration, monocyte population ($CD14^+HLA\text{-}DR^+$) were analyzed by flow cytometry in the lower fraction. The percentage of monocytes is shown in each plot. PBMCs alone or migrating towards MV130 are also shown as control. Data are representative of four independent experiments. (**p <0,01; ***p <0,05 by Wilcoxon test).

Supplemental Table S1: TaqMan Gene expression assay

**Gene**	**Reference**
CCL2	Hs00234140_m1
CCL5	Hs00174575_m1
CSF1	Hs00174164_m1
CXCL12	Hs00171022_m1
CXCL8	Hs00174103_m1
FGF2	Hs00266645_m1
IL1B	Hs01555410_m1
IL6	Hs00985639_m1
LGALS1	Hs00355202_m1
NANOG	Hs02387400_g1
POU5F1(Oct-4)	Hs03005111_g1
PTGS2 (COX2)	Hs00153133_m1
RACK1 (GNB2L1)	Hs00272002_m1
TGFB1	Hs00998133_m1
TLR1	Hs00413978_m1
TLR2	Hs01872448_s1
TLR3	Hs00152833_m1
TLR4	Hs00152939_m1
TLR5	Hs00152825_m1
TLR6	Hs00271977_s1
TLR9	Hs00152973_m1
VEGFA	Hs00900055_m1

Supplemental Table S2. Human and mouse antibodies used for flow cytometry

Human Target Molecule	Clone	Manufacturer
Bcl-xL	H-5	
CD163	GH1/G1	Biolegend
CD19	HIB19	
CD1a	HI149	
CD25	BC96	
CD39	A1	
CD4	OKT4	
CD69	FN50	
CD73	AD2	
CD86	IT2.2	
CD90	5E10	
DC-SIGN	9E9A8	
HLA-AB	W6/32	
HLA-DR	L243	
ICAM1	HCD54	
MMR	15-2	
PDL1	29E.2A3	
PDL2	4F.10C12	
CD14	47-3D6	Inmunostep
CD3	33-2A3	
Bcl-2	Bcl-2/100	BD Biosciences
CD40	5C3	
VCAM1	51-10C9	
CD16	REA423	Miltenyi Biotec
**Mouse Target Molecule**	**Clone**	**Manufacturer**
CD29	HMβ1-1	Biolegend
CD3	17A2	
CD45	30-F11	
CD68	FA-11	
F4/80	BM8	
Gr-1	RB6-8C5	
MHC class II (I-A/I-E)	M5/114.15.2	
Sca-1	D7	
